# Maternal folic acid supplementation and dietary folate intake and congenital heart defects

**DOI:** 10.1371/journal.pone.0187996

**Published:** 2017-11-16

**Authors:** Baohong Mao, Jie Qiu, Nan Zhao, Yawen Shao, Wei Dai, Xiaochun He, Hongmei Cui, Xiaojuan Lin, Ling Lv, Zhongfeng Tang, Sijuan Xu, Huang Huang, Min Zhou, Xiaoying Xu, Weitao Qiu, Qing Liu, Yawei Zhang

**Affiliations:** 1 Gansu Provincial Maternity and Child Care Hospital, Qilihe District, Lanzhou, Gansu Province, China; 2 Yale University School of Public Health, New Haven, Connecticut, United States of America; Oslo universitetssykehus Ulleval, NORWAY

## Abstract

**Background:**

It has been reported that folic acid supplementation before and/or during pregnancy could reduce the risk of congenital heart defects (CHDs). However, the results from limited epidemiologic studies have been inconclusive. We investigated the associations between maternal folic acid supplementation, dietary folate intake, and the risk of CHDs.

**Methods:**

A birth cohort study was conducted in 2010–2012 at the Gansu Provincial Maternity & Child Care Hospital in Lanzhou, China. After exclusion of stillbirths and multiple births, a total of 94 births were identified with congenital heart defects, and 9,993 births without any birth defects. Unconditional logistic regression was used to estimate the associations.

**Results:**

Compared to non-users, folic acid supplement users before pregnancy had a reduced risk of overall CHDs (OR: 0.42, 95% CI: 0.21–0.86, *P*_trend_ = 0.025) after adjusted for potential confounders. A protective effect was observed for certain subtypes of CHDs (OR: 0.37, 95% CI: 0.16–0.85 for malformation of great arteries; 0.26, 0.10–0.68 for malformation of cardiac septa; 0.34, 0.13–0.93 for Atrial septal defect). A similar protective effect was also seen for multiple CHDs (OR: 0.49, 95% CI: 0.26–0.93, *P*_trend_ = 0.004). Compared with the middle quartiles of dietary folate intake, lower dietary folate intake (<149.88 μg/day) during pregnancy were associated with increased risk of overall CHDs (OR: 1.63, 95% CI: 1.01–2.62) and patent ductus arteriosus (OR: 1.85, 95% CI: 1.03–3.32). Women who were non-user folic acid supplement and lower dietary folate intake have almost 2-fold increased CHDs risk in their offspring.

**Conclusions:**

Our study suggested that folic acid supplementation before pregnancy was associated with a reduced risk of CHDs, lower dietary folate intake during pregnancy was associated with increased risk. The observed associations varied by CHD subtypes. A synergistic effect of dietary folate intake and folic acid supplementation was also observed.

## Introduction

Congenital heart defects (CHDs) are among the major birth defects in newborns and affect approximately 4 to 10 per 1,000 live births [1–3]. The CHD prevalence had increased substantially, from 0.6 per 1,000 live births in 1930–1934 to 9.1 per 1,000 live births in 2005–2009 [3]. The disease burden caused by CHDs had reached 224 Disability-adjusted life years (DALYs) per 100,000 persons, accounts for 39.72 percent of all congenital anomalies worldwide based Global Burden of Disease Study on 2010 [4]. In china the prevalence of CHDs are particularly high and become the most frequent type of birth defects with the prevalence of 28.82 per 10,000 persons in 2009 [5]. The etiology of CHDs is poorly understood, and thus the prevention measures have been limited.

Two recent studies reported a reduced rate of CHDs in Quebec (Canada) and Atlanta (USA) over the past years and suggested that folic acid fortification of grain products might play a role [6, 7]. Epidemiological studies that investigated the association between folic acid intake and the risk of CHDs have reached conflicting results. Twelve studies found that folic acid supplementation before and/or during pregnancy reduced the risk of CHDs [8–19], six studies reported no association between folic acid supplements and CHD risk [20–25], while one study found a significantly increased risk of CHDs associated with folic acid supplement use[26]. Three studies investigated dietary folate intake and risk of CHDs and found no association [27–29]. No study has investigated folic acid supplementation and dietary folate intake simultaneously in relation to the risk of CHDs. To further clarify the role of folic acid supplements and dietary folate intake, we analyzed data from a birth cohort study conducted in Lanzhou, China.

## Materials and methods

### Study population

A birth cohort study was conducted at the Gansu Provincial Maternity and Child Care Hospital during 2010–2012 in Lanzhou, China [30, 31]. In brief, after obtaining written consent, eligible pregnant women who came to the hospital for delivery were interviewed by trained study interviewers using a standardized and structured questionnaire to collect the detail information on demographics, residential history, smoking and alcohol consumption, occupational history, medical history, reproductive history, nutrient supplements, and diet. Information on birth outcomes and maternal complications was abstracted from the medical records. A total of 10,542 pregnant women (73.42%) were completed the questionnaire. All study procedures were approved by the Human Investigation Committees at the Gansu Provincial Maternity and Child Care Hospital and Yale University.

**Fig 1:**
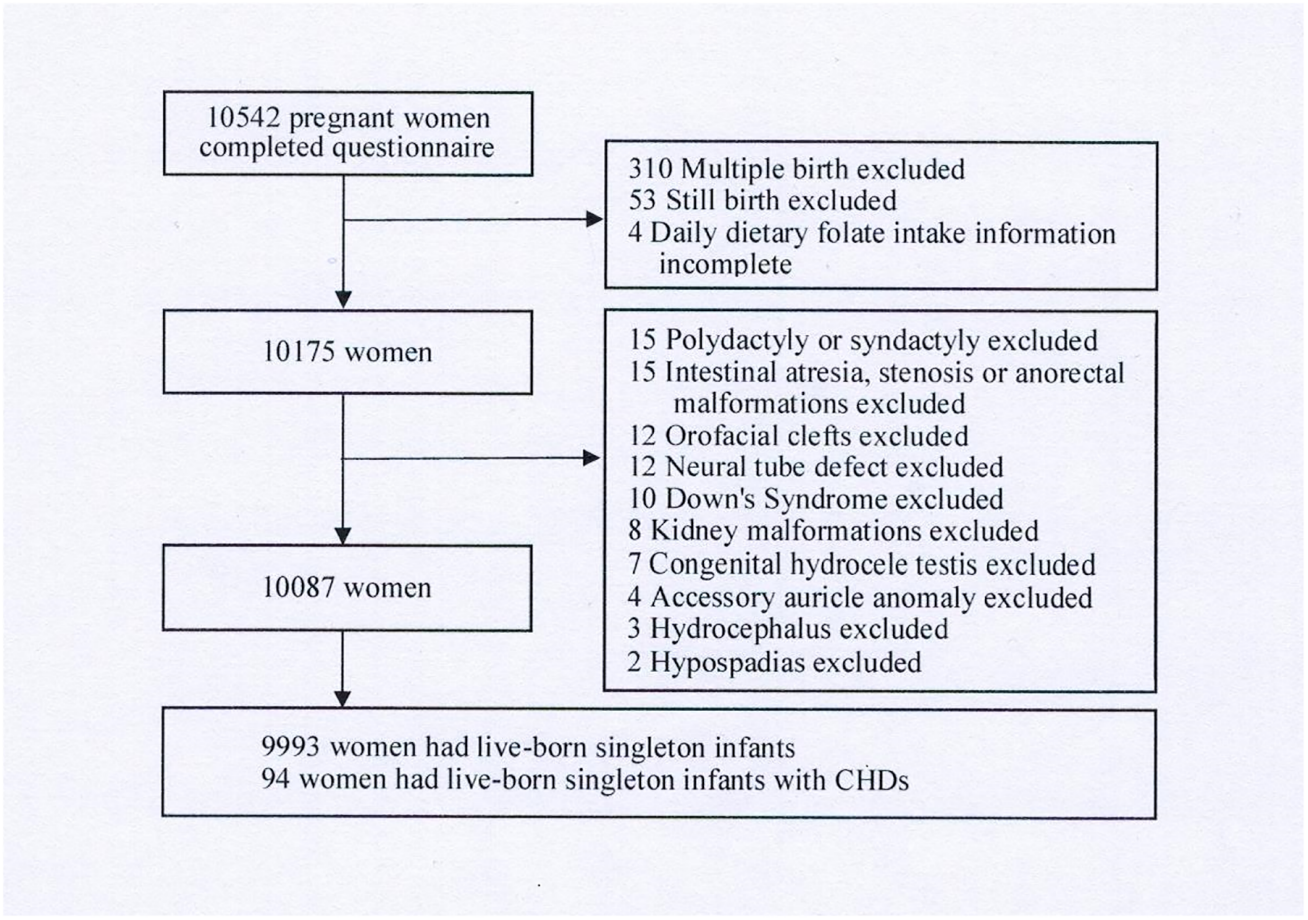
The flow chart of study population selection.

After exclusion of multiple births, stillbirths and/or births with other birth defects (non-CHDs), 10,087 singleton live births were included in the study (As shown in Fig 1).

### Classification of congenital heart defects

Each congenital heart defect was described as “Isolated”, “Multiple” or “Syndrome” based on the defect’s complexity. The isolated CHD category was described either an isolated CHD or a well defined single entity (such as tetralogy of Fallot). The multiple CHD category was described the infants with more than one distinct CHDs (eg, atrial septal defect with patent ductus arteriosus) and without any non-CHDs congenital anomalies. The Syndrome CHD category was described the infants with distinct CHDs associated with any non-CHD congenital anomalies. All of CHDs were classified into three subgroups by International Classification of Diseases (ICD)-10 code (Q20–Q28) and the clinical phenotype: (1) Congenital malformations of great arteries (ICD 10: Q25), including patent ductus arteriosus (PDA), coarctation of aorta, stenosis of pulmonary artery, atresia of pulmonary artery, and other congenital malformations of great arteries. (2) Congenital malformations of cardiac septa (ICD 10: Q21), including atrioventricular septal defects (AVSDs), ventricular septal defects (VSDs), atrial septal defects (ASDs), tetralogy of Fallot, aortopulmonary septal defect, and other congenital malformations of cardiac septa. (3) Other CHDs, including congenital malformations of cardiac chambers and connections, congenital malformations of aortic and mitral valves, other congenital malformations of heart, etc. Among 10,087 individuals, 94 women had live-born singleton infants with CHDs, 43 infants had isolated defect, 46 infants had multiple defect, and other 5 infants had syndrome defect. Among 94 CHDs, 69 were congenital malformations of the great arteries, 58 were congenital malformations of cardiac septa, and 18 were other congenital heart defects.

### Folic acid supplementation and dietary folate intake

The process of data collection on folic acid supplementation and dietary folate intake has been described previously [31, 32]. Briefly, four time periods information about folic acid supplements and dietary folate intake were collected: before conception (12 months before pregnancy), first trimester (1–13 weeks), second trimester (14–27 weeks) and third trimester (>27 weeks). In every period, frequency and duration of folic acid supplement alone and folic acid-containing multivitamins were ascertained. Nonusers were defined as those who never took folic acid supplement alone or folic acid-containing multivitamins before conception and/or during pregnancy, users were defined as those who took folic acid supplement alone or folic acid-containing multivitamins before conception and/or during pregnancy. Dietary information was collected via a semiquantitative food frequency questionnaire (including 39 food items) for the four time periods: before conception (12 months before pregnancy), first trimester (1–13 weeks), second trimester (14–27 weeks) and third trimester (>27 weeks). Daily dietary folate intake for each time period was estimated from the frequency of consumption and portion size of food items using the Chinese Standard Tables of Food Consumption [33]. Daily dietary folate intake during pregnancy was estimated by averaging the intake from three trimesters.

### Statistical analysis

Distributions of sociodemographic and lifestyle characteristics between cases and controls were compared using χ2-tests. Unconditional logistic regression models were used to estimate the odds ratios (*OR*) and 95% confidence intervals (*CI*) for the association of folic acid supplements or dietary folate intake and the risk of CHD and its clinical subtypes. We classified duration of the folic acid supplements into two levels (≤12 weeks, >12 weeks) by midpoint of duration of use, to indicate the low and high levels. Dose response relationship (P for trend) was estimated by including a categorical variable (values 0, 1, 2 representing non-users, low and high level users, respectively). Dietary folate intake was categorized as <25th percentile, 25th to 75th percentile, and ≥75th percentile, based on the distribution of each duration of dietary folate intake among controls. In the final models, we adjusted for potential confounding variables, including maternal age, education level (<college, ≥college), family monthly income per capita (<3000, ≥3000 RMB), employment (never, employed during pregnancy, unemployed during pregnancy), maternal BMI (<18.5, 18.5–23.9, ≥24.0 kg/m^2^), gestational diabetes (yes or no), abortion history (yes or no), parity (primipara, multipara). Additional adjustment for alcohol drinking, active and passive smoking, infant gender, maternal anemia did not result in material changes of the observed associations, thus these covariates were not included in the final models. All analyses were performed using SAS 9.3 software (SAS Institute Inc., Cary, NC, USA) and *P* < 0.05 and 95% CI excluding 1.0 was considered statistically significant.

## Results

Among 10,087 individuals, 94 women had live-born singleton infants with CHDs. Among those infants, 43 had isolated defect, 46 had multiple defects, and 5 had syndromic defects. Specifically, 69 were congenital malformations of the great arteries, 58 were congenital malformations of cardiac septa, and 18 were other CHDs.

As shown in Table 1, compared with mothers whose infants did not have CHDs, mothers of infants with CHDs were more likely to have gestational diabetes, have lower dietary folate intake and family income. Distributions of maternal age, education, unemployment, smoking, alcohol consumption, abortion history, and parity were similar between mothers whose infants with and without CHDs.

**Table 1:** Maternal characteristics of cases and controls, China, 2010–2012.

Characteristics	controls (n = 9993)		cases (n = 94)		P-value
	n	%	n	%	
Maternal age					
<25	1601	98.83	19	1.17	0.084
25–29	3756	99.34	25	0.66	
≥30	4636	98.93	50	1.07	
Highest education level					
< College	6095	98.99	62	1.01	0.239
≥College	3716	99.23	29	0.99	
Missing	182	98.38	3	1.62	
Monthly income per capita(RMB)					
< 3000	5037	98.92	55	1.08	0.039
≥3000	4005	99.33	27	0.67	
Missing	951	98.75	12	1.25	
Maternal employment					
never employ	3271	98.94	35	1.06	0.351
employed during pregnancy	5155	99.06	49	0.94	
unemployed during pregnancy	1567	99.37	10	0.63	
Smoking (passive and active)					
No	8037	99.08	75	0.92	0.876
Yes	1956	99.04	19	0.96	
Alcohol consumption during pregnancy					
No	9973	99.07	94	0.93	0.829*
Yes	20	100.00	0	0.00	
BMI (kg/m^2^)					
<18.5	2379	99.00	24	1.00	0.315
18.5–23.9	6561	99.15	56	0.85	
≥24.0	1053	98.69	14	1.31	
Gestational diabetes					
No	9898	99.12	88	0.88	<0.001
Yes	95	94.06	6	5.94	
Abortion history					
No	8685	99.02	86	0.98	0.189
Yes	1308	99.39	8	0.61	
Parity					
Primipara	7212	99.11	65	0.89	0.515
Multipara	2781	98.97	29	1.03	
Folic acid supplement					
Nonusers	2676	98.78	33	1.22	0.069
Users	7317	99.17	61	0.83	
Dietary folate intake(μg/day)					
<25th percentile	2491	98.65	34	1.35	0.043
25th-75th percentile	5002	99.21	40	0.79	
≥75th percentile	2500	99.21	20	0.79	

Calculated by χ^2^ analysis without accounting for missing data

* Fisher exact test

Compared to nonusers, ever folic acid supplement users did not experience a significantly reduced risk of CHDs (OR: 0.81, 95% CI: 0.51–1.29, Table 2). While a suggestive reduced risk of CHDs was observed for longer term users (OR = 0.57, 95%CI: 0.34–0.95), however, the protective effect was attenuated after additional adjustment for dietary folate intake (OR: 0.64, 95%CI: 0.38–1.08). After stratifying by time periods of use of folic acid supplements, significantly protective effect was observed for those who took supplements before pregnancy (OR: 0.42, 95% CI: 0.21–0.86) with a significant dose-response for duration of use (*P* for trend = 0.025). No significant association was observed among women who took supplements during pregnancy. Compared with the middle quartiles (25th to 75th) of dietary folate intake, an increased risk was observed for those who were in the lowest quartile of dietary folate intake during pregnancy (OR: 1.63, 95% CI: 1.01–2.62). No significant association was observed between dietary folate intake before pregnancy and risk of CHDs.

**Table 2:** Associations between folate intake and the risk of overall congenital heart defects, China, 2010–2012.

Folic acid/folate intake duration	Controls (n = 9993)	Overall Congenital Heart Defects		
		Cases(n = 94)	*OR*^a^ (*95% CI*)	*OR*^b^ (*95% CI*)
Folic acid supplement				
Nonusers	2676	33	1.00	1.00
Users	7317	61	0.73(0.46–1.14)	0.81(0.51–1.29)
≤12 weeks	2385	28	1.00(0.60–1.68)	1.11(0.66–1.89)
>12 weeks	4932	33	0.57(0.34–0.95)	0.64(0.38–1.08)
*P* for trend			0.009	0.053
Before pregnancy	3081	14	0.38(0.19–0.76)	0.42(0.21–0.86)
≤8 weeks	1389	6	0.36(0.14–0.89)	0.40(0.16–1.03)
>8 weeks	1692	8	0.40(0.17–0.93)	0.43(0.18–1.02)
*P* for trend			0.005	0.025
During pregnancy	6814	60	0.77(0.49–1.20)	0.87(0.55–1.38)
≤12 weeks	2511	29	0.95(0.57–1.58)	1.06(0.63–1.79)
>12 weeks	4303	31	0.63(0.37–1.06)	0.71(0.42–1.22)
*P* for trend			0.035	0.146
Dietary folate intake(μg/day)				
Before pregnancy				
<115.97	2498	32	1.42(0.88–2.27)	1.39(0.86–2.23)
115.97, 221.03	4997	42	1.00	1.00
≥221.03	2498	20	0.96(0.56–1.64)	0.93(0.54–1.59)
*P* for trend			0.157	0.152
During pregnancy				
<149.88	2498	34	1.67(1.04–2.67)	1.63(1.01–2.62)
149.88, 266.35	4990	39	1.00	1.00
≥266.35	2505	21	1.08(0.63–1.86)	1.06(0.62–1.81)
*P* for trend			0.099	0.105

^a^ Adjusted for maternal age, education level, family monthly income per capita, employment, maternal BMI, diabetes, abortion history, Parity.

^b^ Adjusted for maternal age, education level, family monthly income per capita, employment, maternal BMI, diabetes, abortion history, Parity, dietary folate intake or folic acid supplement.

After stratifying by CHD subtypes, similar associations with folic acid supplements were observed for congenital malformation of great arteries and congenital malformation of cardiac septa (Table 3). Lower dietary folate intake during pregnancy was associated with a suggestive increased risk of congenital malformation of great arteries (OR: 1.71 95% CI: 0.97–2.99) but not congenital malformation of cardiac septa (OR: 1.24, 95% CI: 0.67–2.32).

**Table 3:** Associations between folate intake and the risk of subtypes of congenital heart defects, China, 2010–2012.

Folic acid/folate intake duration	Controls (n = 9993)	Congenital malformation of great arteries			Congenital malformation of cardiac septa		
		Cases(n = 69)	*OR*^a^ (*95% CI*)	*OR*^b^ (*95% CI*)	Cases(n = 58)	*OR*^a^ (*95% CI*)	*OR*^b^ (*95% CI*)
Folic acid supplement							
Nonusers	2676	27	1.00	1.00	24	1.00	1.00
Users	7317	42	0.60(0.36–1.01)	0.67(0.40–1.14)	34	0.54(0.31–0.95)	0.62(0.35–1.10)
≤12 weeks	2385	20	0.88(0.48–1.59)	0.97(0.53–1.78)	15	0.75(0.39–1.45)	0.84(0.43–1.65)
>12 weeks	4932	22	0.45(0.25–0.82)	0.50(0.27–0.93)	19	0.43(0.23–0.82)	0.50(0.26–0.96)
*P* for trend			0.003	0.015		0.004	0.027
Before pregnancy	3081	10	0.32(0.14–0.73)	0.37(0.16–0.85)	7	0.24(0.09–0.61)	0.26(0.10–0.68)
≤8 weeks	1389	4	0.29(0.10–0.87)	0.34(0.11–1.05)	3	0.23(0.06–0.80)	0.25(0.07–0.90)
>8 weeks	1692	6	0.35(0.13–0.93)	0.40(0.15–1.06)	4	0.25(0.08–0.80)	0.27(0.08–0.87)
*P* for trend			0.006	0.029		0.003	0.011
During pregnancy	6814	41	0.63(0.38–1.06)	0.71(0.42–1.21)	33	0.57(0.33–1.00)	0.66(0.37–1.17)
≤12 weeks	2701	22	0.85(0.48–1.53)	0.95(0.52–1.72)	16	0.70(0.37–1.35)	0.80(0.41–1.55)
>12 weeks	4113	19	0.47(0.25–0.88)	0.53(0.28–1.01)	17	0.47(0.24–0.92)	0.55(0.28–1.09)
*P* for trend			0.007	0.033		0.012	0.064
Dietary folate intake(μg/day)							
Before pregnancy							
<115.97	2498	22	1.26(0.72–2.19)	1.21(0.69–2.12)	20	1.55(0.84–2.85)	1.48(0.80–2.74)
115.97, 221.03	4997	32	1.00	1.00	24	1.00	1.00
≥221.03	2498	15	0.94(0.50–1.75)	0.88(0.47–1.65)	14	1.16(0.59–2.26)	1.08(0.55–2.12)
*P* for trend			0.334	0.319		0.377	0.368
During pregnancy							
<149.88	2498	25	1.78(1.02–3.11)	1.71(0.97–2.99)	18	1.31(0.71–2.43)	1.24(0.67–2.32)
149.88, 266.35	4990	27	1.00	1.00	26	1.00	1.00
≥266.35	2505	17	1.27(0.69–2.35)	1.22(0.66–2.26)	14	1.08(0.56–2.10)	1.02(0.53–1.99)
*P* for trend			0.215	0.227		0.562	0.595

^a^ Adjusted for maternal age, education level, family monthly income per capita, employment, maternal BMI, diabetes, abortion history, Parity.

^b^ Adjusted for maternal age, education level, family monthly income per capita, employment, maternal BMI, diabetes, abortion history, Parity, dietary folate intake or folic acid supplement

Associations between folate intake and risk of both patent ductus arteriosus (PDA) and atrial septal defect (ASD) were shown in Table 4. Compared to nonusers, a suggestive reduced risk of PDA was observed for longer term folic acid supplement users (OR 0.55, 95% CI: 0.29–1.04) with a significant dose-response (*P* for trend = 0.041), and a significantly reduced risk of ASD was observed for those who took supplements before pregnancy (OR: 0.34, 95% CI: 0.13–0.93). Compared with the middle quartiles of dietary folate intake, lower dietary folate intake during pregnancy increased the risk of PDA (OR: 1.85, 95% CI: 1.03–3.32), but not ASD (OR: 1.44, 95% CI: 0.72–2.87).

**Table 4:** Associations between folate intake and the risk of patent ductus arteriosus and atrial septal defect, China, 2010–2012.

Folic acid/folate intake duration	Controls (n = 9993)	Patent ductus arteriosus			Atrial septal defect		
		Cases(n = 65)	*OR*^a^ (*95% CI*)	*OR*^b^ (*95% CI*)	Cases(n = 46)	*OR*^a^ (*95% CI*)	*OR*^b^ (*95% CI*)
Folic acid supplement							
Nonusers	2676	24	1.00	1.00	18	1.00	1.00
Users	7317	41	0.67(0.39–1.14)	0.75(0.43–1.29)	28	0.59(0.31–1.10)	0.67(0.35–1.27)
≤12 weeks	2385	20	0.98(0.53–1.79)	1.08(0.58–2.01)	12	0.77(0.37–1.63)	1.87(0.41–1.87)
>12 weeks	4932	21	0.49(0.27–0.92)	0.55(0.29–1.04)	16	0.48(0.23–0.98)	0.55(0.26–1.15)
*P* for trend			0.011	0.041		0.022	0.082
Before pregnancy	3081	9	0.36(0.15–0.85)	0.42(0.17–1.00)	7	0.31(0.11–0.82)	0.34(0.13–0.93)
During pregnancy	6814	40	0.71(0.41–1.21)	0.46(0.42–1.37)	27	0.61(0.33–1.15)	0.70(0.37–1.35)
Dietary folate intake(μg/day)							
Before pregnancy							
<115.97	2498	20	1.16(0.65–2.07)	1.13(0.63–2.02)	15	1.55(0.78–3.10)	1.50(0.75–3.00)
115.97, 221.03	4997	31	1.00	1.00	19	1.00	1.00
≥221.03	2498	14	0.91(0.48–1.73)	0.87(0.46–1.66)	12	1.27(0.61–2.64)	1.20(0.58–2.51)
*P* for trend			0.469	0.451		0.594	0.581
During pregnancy							
<149.88	2498	24	1.91(1.07–3.40)	1.85(1.03–3.32)	15	1.50(0.76–2.99)	1.44(0.72–2.87)
149.88, 266.35	4990	24	1.00	1.00	20	1.00	1.00
≥266.35	2505	17	1.44(0.77–2.71)	1.40(0.74–2.64)	11	1.12(0.53–2.36)	1.07(0.50–2.25)
*P* for trend			0.312	0.321		0.431	0.451

^a^ Adjusted for maternal age, education level, family monthly income per capita, employment, maternal BMI, diabetes, abortion history, Parity.

^b^ Adjusted for maternal age, education level, family monthly income per capita, employment, maternal BMI, diabetes, abortion history, Parity, dietary folate intake or folic acid supplement

We further analyzed the data by multiple CHDs and isolated CHDs (Table 5). Compared with nonusers, significantly protective effect of folic acid supplement use was observed for multiple CHDs (OR 0.49, 95% CI 0.26–0.93) with a significant dose-response (*P* for trend = 0.004). The associations were similar for different time periods of use of folic acid supplements. No significant association was observed between folic acid supplement use and isolated CHD. A borderline significant increase in risk of multiple CHDs was found among women who had lower dietary folate intake before pregnancy (OR: 1.97, 95% CI 0.98–3.97). Dietary folate intake was not associated with isolated CHD.

**Table 5:** Associations between folate intake and the risk of multiple and isolated congenital heart defects, China, 2010–2012.

Folic acid/folate intake duration	Controls (n = 9993)	Multiple CHDs			Isolated CHDs		
		Cases(n = 46)	*OR*^a^ (*95% CI*)	*OR*^b^ (*95% CI*)	Cases(n = 43)	*OR*^a^ (*95% CI*)	*OR*^b^ (*95% CI*)
Folic acid supplement							
Nonusers	2676	22	1.00	1.00	10	1.00	1.00
Users	7317	24	0.41(0.22–0.77)	0.49(0.26–0.93)	33	1.32(0.63–2.75)	1.39(0.66–2.99)
≤12 weeks	2385	12	0.66(0.32–1.35)	0.76(0.36–1.59)	14	1.62(0.71–3.69)	1.70(0.74–3.89)
>12 weeks	4932	12	0.29(0.14–0.61)	0.34(0.16–0.74)	19	1.13(0.50–2.53)	1.19(0.53–2.69)
*P* for trend			0.000	0.004		0.961	0.831
Before pregnancy	3081	7	0.29(0.11–0.76)	0.36(0.13–0.94)	7	0.55(0.19–1.62)	0.53(0.18–1.56)
During pregnancy	6814	23	0.43(0.23–0.81)	0.51(0.27–0.98)	33	1.41(0.67–2.93)	1.49(0.71–3.12)
Dietary folate intake(μg/day)							
Before pregnancy							
<115.97	2498	18	2.11(1.05–4.22)	1.97(0.98–3.97)	14	1.17(0.59–2.30)	1.18(0.60–2.33)
115.97, 221.03	4997	16	1.00	1.00	22	1.00	1.00
≥221.03	2498	12	1.45(0.68–3.10)	1.31(0.61–2.82)	7	0.67(0.28–1.58)	0.69(0.29–1.62)
*P* for trend			0.234	0.232		0.299	0.304
During pregnancy							
<149.88	2498	16	1.71(0.85–3.42)	1.57(0.78–3.16)	17	1.91(0.96–3.78)	1.94(0.98–3.85)
149.88, 266.35	4990	18	1.00	1.00	17	1.00	1.00
≥266.35	2505	12	1.32(0.63–2.78)	1.22(0.58–2.56)	9	1.11(0.49–2.52)	1.14(0.50–2.58)
*P* for trend			0.412	0.451		0.174	0.172

^a^ Adjusted for maternal age, education level, family monthly income per capita, employment, maternal BMI, diabetes, abortion history, Parity.

^b^ Adjusted for maternal age, education level, family monthly income per capita, employment, maternal BMI, diabetes, abortion history, Parity, dietary folate intake or folic acid supplement

A joint effect between folic acid supplement use and dietary folate intake on the risk of CHDs were analyzed. However, there was no significant interaction between folic acid supplementation and dietary folate intake on the risk of CHDs (data not shown). Potential effect modifications of maternal age on the associations between folate intake and risk of CHDs were also analyzed and didn’t observe significant findings (data not shown). Additionally, we observed no interaction between gestational diabetes and folic acid supplement use and dietary folate intake (P for interaction = 0.38 and 0.09, respectively).

## Discussion

Our study suggested that folic acid supplements before pregnancy were associated with a reduced risk of CHDs and the reduced risk varied by CHD subtypes. Our study also suggested that lower dietary folate intake during pregnancy increased the risk of CHDs and the risk varied by CHD subtype. A suggestive joint effect between folic acid supplement and dietary folate intake before pregnancy on the risk of CHD was also observed in our study.

There is biologic plausibility underlying the association between folate and CHD. Accumulating evidence suggests that inadequate folate status prevent the re-methylation of 5-methyl-tetrahydrofolate and lead to the accumulation of homocysteine[34]. Excessive homocysteine results in abnormalities of cardiac neural crest cell migration, differentiation, dispersal, and cell cycle progression, which disrupt the development of cardiac neural crest and lead to the occurrence of CHDs [35, 36]. Adequate folate is necessary for normal development of cardio system of the fetus.

Earlier epidemiological researches investigating the associations between folic acid supplements and the risk of CHDs provided inconsistent results. Five case-control studies [13, 20, 23, 24, 26] reported that take folic acid/ multivitamin supplements had no association with overall CHDs, other four studies [9, 10, 12, 16] reported that folic acid/ multivitamin supplementation could reduce the risk of overall CHDs which consistent with our results. Li et al [16] conducted a hospital based case-control study (2010–2011) in Chengdu (China) including 358 cases and 422 controls and found that pregnant woman who reported multivitamin (containing folic acid) supplementation during the period from three months prior to conception to two months after conception were less likely to have offspring with CHDs (OR = 0.52, 0.34–0.78). Botto et al [10] carried out a population based case-control study in Atlanta including 494 cardiac defects cases and 1610 controls based on Atlanta Birth Defects Research (1968–1980), which found that pregnant woman who regular use of multivitamins from three months before pregnancy through the first three months of pregnancy cut by almost one-quarter risk of overall CHDs(OR = 0.76, 0.60–0.97). Czeizel et al [12] conducted a cohort controlled trial study(1993–1996) in Budapest (Hungary), which found that woman who take multivitamin (containing folic acid) supplementation from one month before conception to three month after conception reduced the risk of overall CHDs(31/3056 vs 50/3056, OR = 0.60, 0.38–0.96). Czeizel [9] carried out a randomized controlled trial (1984–1991) in Budapest (Hungary) to evaluate the efficacy of trace element supplementation in reduction of the occurrence of neural tube defects, which also found that woman who take multivitamin (containing folic acid) supplementation during three month before pregnancy reduced the occurrence of CHDs. However, when assessed the association between folic acid supplementation and CHDs, none of these studies considered the effect of dietary folate intake on the associations between folic acid supplementation and CHDs. Our results showed that the associations of folic acid supplementation with CHDs attenuated when dietary folate intake were included in the model as well as vice versa.

Our study found that longer duration of supplementation had a beneficial effect on reduction of overall CHDs risk. Only one early study [16] explored the association by duration of maternal folic acid supplementation and the results were consistent with our study.

Our study found significant associations between folic acid supplementation and certain subtypes of CHDs, including congenital malformation of great arteries, congenital malformation of cardiac septa, ASD, and multiple CHDs, which were consistent with several previous studies [10, 13, 14, 18], but not all [9, 12, 26]. Botto et al[10] found that regular use of folic acid containing multivitamins from three months before pregnancy through the first three months of pregnancy was associated with a 41% reduced risk of cardiac septal defects, but not ASD. Van-Beynum et al.[13] found a 38% reduced risk of septal defects and a 46% reduced risk of ASD associated with folic acid supplement intake from 4 weeks before conception to 8 weeks after conception, but no association with AVSD and PDA. Bean et al [14] reported that lack of maternal folic acid supplementation before and during pregnancy increased the risk of septal defects in infants. Vereczkey et al [18] found a 49% reduced risk of AVSD associated with folic acid supplements from the preconception period to the first trimester of pregnancy. Leirgul et al [26] reported an increased risk for isolated septal defects if mothers used folic acid supplements before and during pregnancy. Czeizel et al.[9, 12] found no significant association was exist between multiple CHDs and folic acid supplements use. Variations in study populations (American, Hungarian, Dutch, Norwegians, Chinese), the time for initiating supplementation (before and/or during pregnancy) may have contributed to the inconsistent results.

Our study also found lower dietary folate intake (<149.88 μg/day) increased the risk of CHDs and PDA subtype compared with the middle quartiles (25th to 75th) during pregnancy. Few early studies investigated dietary folate intake and risk of CHDs and have reached inconsistent results [27–29]. Smedts et al. [28] reported lower dietary folate intake(<165 μg/day) was not associated with the risk of CHDs. Verkleij-Hagoort et al. [29] also reported no significant association between lower dietary folate intake (<117.6 μg/day) and the risk of CHDs. Shaw et al [27] reported lower dietary folate intake(<293.8 μg/day) associated with increased risk of d-transposition of great arteries but not tetralogy of Fallot. It is challenge to make comparison among these studies due to different cut-off levels of dietary folate intake being used by different studies. In addition, background dietary folate intake might also varied across populations.

Strengths and limitations should be considered when interpreting the study results. Our study collected detailed information on both folic acid supplementation and dietary folate intake, which allowed us to control for each other. While many important confounding factors have been adjusted for, potential residual confounding cannot be entirely ruled out. Information on dietary intake and folic acid supplements were collected through in-person interview at delivery, potential recall bias might play a role. However, the relationships between folic acid supplementation, dietary folate intake, and risk of CHDs have not been well-established, and were unlikely to be known by the general public. Therefore, if there was any recall bias it was likely to be non-differential and resulted in underestimation of the observed associations. The statistical power for CHD subtypes were limited due to the relatively small sample size. While our study was a hospital-based and might affect the generalizability, the prevalence of CHDs (9.3 per 1000 live births) in our study population was comparable to the reported prevalence in various Chinese populations [37–40].

In conclusion, our study results suggest that folic acid supplementation before pregnancy reduces the risk of CHDs and lower dietary folate intake during pregnancy increases the risk of CHDs. Future larger studies with greater statistical power are warranted to elucidate the associations by CHD subtypes and identify women who would benefit the most from folic acid supplements. Finally, our findings have important public health implications and may facilitate the acceptance of taking folic acid supplements during preconception and pregnancy and the increase in dietary folate intake during pregnancy.
